# Satisfaction with emergency obstetric and new born care services among clients using public health facilities in Jimma Zone, Oromia Regional State, Ethiopia; a cross sectional study

**DOI:** 10.1186/s12884-016-0877-0

**Published:** 2016-04-25

**Authors:** Alemayehu Kumsa, Gurmessa Tura, Aderajew Nigusse, Getahun Kebede

**Affiliations:** John Snow Research & Training Institute Inc./the Last Ten Kilometres (JSI/L10K) Project, Jimma, Ethiopia; Department of Population and Family Health, College Of Health Sciences, Jimma University, Jimma, Ethiopia; Institute of Public Health, College of Medicine and Health Sciences, University of Gondar, Gondar, Ethiopia

**Keywords:** Clients’ satisfaction, Emergency obstetric and new born care, Public health facilities, Jimma zone, Ethiopia

## Abstract

**Background:**

The 2005 report of United Nations Millennium Project of Transforming Health Systems for women and children concluded that universal access to Emergency Obstetric and New born Care could reduce maternal deaths by 74 %. Even though some studies investigated quality of Emergency Obstetric and New born Care in different parts of the world, there is scarcity of data regarding this issue in Ethiopia, particularly in Jimma zone. Therefore, the aim of this study was to assess satisfaction with Emergency Obstetric and new born Care services among clients using public health facilities in Jimma zone, Southwest Ethiopia.

**Methods:**

A facility-based cross sectional study was conducted in Jimma Zone from April 01–30, 2014. The data were collected by interviewing 403 clients, who gave birth in the past 12 months prior to data collection in 34 randomly selected public health facilities. The collected data were entered by using Epi-info version 3.5.4 and analysed using SPSS version 20.0. Linear regression analysis was done to ascertain the association between covariates and the outcome variable, and finally the results were presented using frequency distribution tables, graphs and texts.

**Results:**

The overall mean client satisfaction with Emergency Obstetric and New born Care services in this study was 79.4 %; 95 % CI (75 %, 83 %). The result of linear regression analysis revealed that a unit decrease in satisfaction to availability of drugs and equipment, decreased overall clients’ satisfaction by 0.23 unit 95 % CI (0.15, 0.31).

**Conclusions:**

The level of clients’ satisfaction with Emergency Obstetric and New born Care services was low in the study area. Factors such as availability of essential equipment and drugs, health workers’ communication, health care provided, and attitude of health workers had positive association with client satisfaction with Emergency Obstetric and New born Care services. This in turn could affect utilization of Emergency Obstetric and New born Care services and play a role in contribution to maternal and new born mortality. Therefore, the efforts of health facilities leaders and health care providers towards improvement of quality of care could contribute more for better maternal satisfaction.

## Background

Emergency Obstetric and Newborn Care (EmONC) refers to a set of life saving interventions or signal functions used to treat direct obstetric complications that make up approximately 70–80 % of maternal deaths globally. Basic EmONC facilities are expected to provide the following seven services: administration of Parenteral antibiotics; Parenteral oxytocic drugs; Parenteral anticonvulsants for pre-eclampsia; manual removal of retained placenta; removal of retained products of conception; assisted vaginal delivery (vacuum extraction or forceps delivery) and neonatal resuscitation with bag or mask. Comprehensive EmONC facilities are expected to provide caesarean section and blood transfusion in addition to those services provided by the basic EmONC [1].

Despite the medical and public health advances of the past century, globally each year more than 287000 women die from complications related to pregnancy and child birth, and approximately 7.6 million children, including 3.1 million new born die from diseases that are preventable or treatable with existing interventions [2].

At the country level, Ethiopia with 9000 maternal deaths is one of the top ten countries contributed for 60 % of the global maternal deaths in 2010 [3]. One in every 17 Ethiopian children dies before the first birthday while one in every 11 children dies before the fifth birthday. Similarly, neonatal mortality rate is 37 deaths per 1,000 live births, post-neonatal mortality rate is 22 deaths per 1,000 live births, and the prenatal mortality rate is 46 per 1,000 pregnancies [4].

Overall, an estimated 15 % of pregnant women are expected to experience serious obstetric complications [1]. The estimated average interval between onset of major obstetric complications and death in the absence of medical interventions is 2 h for postpartum haemorrhage, 12 h for ante-partum haemorrhage, 1 day for ruptured uterus, 2 days for eclampsia, 3 days for obstructed labour and 6 days for infection [5].

Moreover, the 2011 Ethiopian Demographic and Health Survey (EDHS) showed that only 10 % of births in the past five years were delivered by a skilled provider. Postnatal care is extremely low- 9 in 10 mothers with live birth received no postnatal care and only 5 % of mothers received postnatal care within the critical first two days after delivery [4].

Client satisfaction is of prime importance as a measure of the quality of medical services because it gives information about provider’s success at meeting those rightful client values and expectations. The measurement of satisfaction is, therefore, an important tool for research, administration and planning [6]. However, epidemiologic data concerning this issue is critically lacking in Ethiopia, particularly in Jimma zone. Thus, the results of this study will be helpful in providing baseline information upon which health services most needed for pregnant women and new-borns can be tailored and strengthened in future. Further, this information is critical for planning purposes, resource allocation and the support of human resource development (training, deployment, and retention). It will also help to develop advocacy tools that will be useful for negotiations with donors, and to narrow the information gaps and make local planning more evidence based. Therefore, it is necessary to conduct this study on client satisfaction with EmONC services among clients using public health facilities in the specified study area.

## Methods

### Study design and setting

Facility-based cross-sectional study was conducted from April 1–30, 2014 to determine clients’ satisfaction with emergency obstetric and new born care services among clients using public health facilities in Jimma Zone, Ethiopia. Jimma zone has 17 districts and two town administrations, each being administratively responsible to the Zone. The total population of Jimma zone projected for the year 2014 from 2007 census was 3,030,740; of which, 94 % are rural residents and the rest are urban. The expected women of reproductive age group were 669,794 and the expected number of pregnant women in the zone was 116,077 in the year 2014. There are 4 public hospitals and 100 public health centres providing delivery service in the zone.

### Sample size determination and sampling technique

The sample size of clients (mothers) was determined by using a single population proportion formula with *P* = 0.61 (proportion of mother’s satisfaction for delivery services in Amhara region referral hospitals) [7], level of significance 5 % (α = 0.05), margin of error 5 % (*d* = 0.05), and 10 % non-response rate. Accordingly, a total of 403 mothers who gave births in the past 12 months prior to the study period were the sample participants of the study.

A handbook for monitoring emergency obstetric care [1] recommends, if there are 25 or fewer hospitals, to study all of them, and if there are more than 25 hospitals, to select a sub-set as many as possible that should represent at least 30 %; and for lower-level facilities including health centres, if there are 100 or fewer, to study all of them, and if there are more than 100, to select a sub-set as many as possible that should represent at least 30 %. Visiting all the health facilities in Jimma zone was difficult in terms of cost and availability of human power and time. Therefore, in order to minimize bias the existing health facilities were broadly listed according to facility type, namely, hospitals and health centres. Then, by using simple random sampling method 30 (30 %) health centres and all the four hospitals (100 %), thus a total of 34 health facilities, were selected for the study. Finally, the women who gave births in those facilities in the past 12 months prior to the study period were interviewed conveniently in proportionate to average monthly delivery load.

### Data collection tool validation and data collection procedures

A structured interview administered questionnaire was developed in English language and translated to Afan Oromo (local language) and back to English by independent language experts to keep the consistency. Training was given for data collectors about techniques of data collection and briefed on each question contained in the data collection tool. As well, the data collection instrument has been validated through conducting pre-test in health facilities nearby Jimma zone prior to the real data collection time. Finally, the required data were collected by interviewing women who were getting delivery services from health facilities using Afan Oromo version questionnaire.

### Measurement

The questionnaire was composed of two parts: (i) Socio-demographic variables and (ii) 19 satisfaction related variables which came up with a high internal consistency (Cronbach’s alpha = 0.756). A five point Likert scale, coded as 1 = completely dissatisfied, 2 = somewhat dissatisfied, 3 = neither satisfied nor dissatisfied, 4 = somewhat satisfied and 5 = completely satisfied was used to rate satisfaction with various aspects of health care. Satisfaction variables were grouped into six dimensions related to: (i) health facilities’ physical environment (8 questions), (ii) health workers’ communication (3 questions), (iii) health care provision (3 questions), (iv) health workers’ attitude (3 questions), and (v) overall satisfaction of clients (2 questions). The overall of clients’ satisfaction was measured with the summation of satisfaction levels of the independent variables. After obtaining the mean score of all independent satisfaction variables, the median of the result was calculated. Those who scored above the median were categorized as fully satisfied and those below the median were categorized as fully dissatisfied with the overall services.

### Operational definitions

Emergency Obstetric and Newborn Care is a set of critical life saving functions commonly called signal functions provided by a health facility, 24 h a day, 7 days a week.

### Data processing and analysis

Data were entered by using data entry software Epi Info version 3.5.4 and analysed using SPSS version 20.0. Frequencies and percentages were computed to describe major findings of the study. Linear regression analysis was done to identify variables which had association with clients’ satisfaction with EmONC services independently. Then, variables which had *P*-values < 0.2 in linear regression analysis were entered in to multivariable linear regression model for controlling possible effects of confounders.

Thus, the association between covariate and the outcome variable was ascertained based on standardized Beta with 95 % Confidence Interval (CI) and *P*-values. A *P*-value of ≤0.05 was considered to identify factors associated with clients’ satisfaction with EmONC services, and finally data were presented using tables and texts.

## Ethical consideration

The study was conducted after official review and approval of the proposal by the Ethical Review Committee (ERC) of the  College of Health Sciences of Jimma University. Letters of cooperation were also obtained from Jimma Zonal Health Department and each District Health Office. Besides, verbal informed consent was obtained from each study participant after clearly explaining the objective of the study.

## Results

### Socio-demographic and maternity related characteristics

From the total of 403 sampled mothers, 399 were interviewed, forming a response rate of 99 %.

Most 157 (39.3 %) of the clients were found in the age group of 20–24years followed by 107 (26.8 %) in the age group of 25–29 years. Significant proportion of the clients 181 (45.4 %) cannot read and write, while 104 (26 %) of the clients attained secondary school or above. Monthly income of most of the clients 281 (70.4 %) was below 600 Ethiopian Birrs (ETB) (Tables 1 and 2).

**Table 1:** Socio demographic Characteristics of the respondents in Jimma zone public health facilities, 2014 (*n* = 399)

Age in years	Frequency (%)	Educational category	Frequency (%)
<19	36 (9 %)	Cannot read and write	181 (45.4 %)
20–29	264 (66.2 %)	Primary (Grade1–8)	114 (28.6 %)
30–39	95 (23.8 %)	Secondary (Grade9–12)	64 (16.0 %)
>39	4 (1.0 %)	Above Secondary	40 (10.0 %)
Residence		Monthly Income	
Urban	131 (32.8 %)	<600ETB	281 (70.4 %)
Rural	268 (67.2 %)	≥600ETB	118 (29.6 %)

**Table 2:** Past obstetric history of the respondents in Jimma zone public health facilities, 2014 (*n* = 399)

Pregnancy status	Frequency (%)	Delivery attended by	Frequency (%)
Wanted	303 (75.9 %)	Physician	40 (10 %)
Unwanted	96 (24.1 %)	Midwives/Nurse	270 (67.7 %)
How Respondents Visited the Facility		Unknown	89 (22.3 %)
Came after referral	94 (23.5 %)	Delivery Outcome	
Relatives recommendation	76 (19.0 %)	Alive	370 (92.7 %)
Came due to emergency	16 (4.0 %)	Died	29 (7.3 %)
Came up on personal decision	189 (47.4 %)	Mode of Delivery	
Others	24 (6.0 %)	Spontaneous Vaginal Delivery	370 (92.7 %
Mode of Transportation		Instrumental Delivery	8 (2.0 %)
Stretcher	181 (45.4 %)	Elective Caesarean Section	3 (0.8 %)
Private vehicle	18 (4.5 %)	Emergency C/S	18 (4.5 %)
Public Transport	69 (17.3 %)	Hours of Stay in facility	
Others (Ambulance, etc.)	131(32.8 %)	<1 h	125 (31.3 %)
Treatment Fee		13–24 h	178 (44.6 %)
Paying	53 (11.3 %)	25–48 h	49 (12.3 %)
Free	346 (86.7 %)	>48 h	47 (11.8 %)

### Clients’ satisfaction with EmONC services

**Fig. 1:**
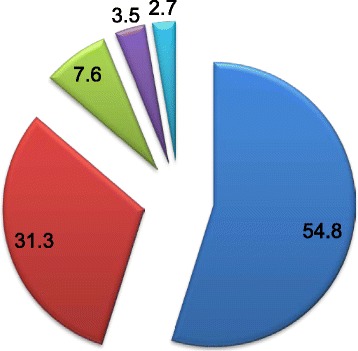
Percentage level of satisfaction to obstetric services in Jimma zone public health facilities, April 2014 (*n* = 399). *Blue color*- represents percentage of completely satisfied clients. *Red color*-represents percentage of somewhat satisfied clients. *Olive Green*-represents percentage of neither satisfied nor dissatisfied clients. *Purple color*-represents percentage of somewhat dissatisfied. *Aqua color*- represents percentage of completely dissatisfied clients

The level of clients’ satisfaction as measured by composite score of  19 items in decreasing orders were: completely satisfied 219 (54.8 %), somewhat satisfied 125 (31.3 %), neither satisfied nor dissatisfied 30 (7.6 %), somewhat dissatisfied 14 (3.5 %) and completely dissatisfied 11 (2.7 %) (Fig. 1). In this study the overall mean clients’ satisfaction with EmONC services was 79.4 %. The detail of respondents' satisfaction with each of the 19 items is presented in Table 3.

**Table 3:** Proportion of respondents' satisfaction with each of the 19 EmONC services satisfaction measuring items and their mean satisfaction score in Jimma zone public health facilities, April 2014 (*n* = 399)

Aspects of care received during stay in maternity ward	Frequency of perceived satisfaction in number (%)				
	Completely Dissatisfied	Somewhat Dissatisfied	Neither Satisfied Nor Dissatisfied	Somewhat Satisfied	Completely Satisfied
Health Facilities Physical Environment					
Availability of adequate number of health staffs	0 (0)	24 (6.0)	29 (7.3)	148 (37)	198 (49.6)
Availability of water, hand washing & toilet facilities	4 (1.0)	5 (1.3)	13 (3.3)	166 (42)	211 (52.9)
Availability of examination equipment	1 (.3)	17 (4.3)	47 (11.8)	187 (47)	147 (36.8)
Availability of drugs and supplies	12 (3.0)	4 (1.0)	19 (4.8)	152 (38)	212 (53.1)
Distance from home to the health facility	19 (4.8)	47 (12)	78 (19.5)	149 (37)	106 (26.6)
Availability of transportation & communication	25 (6.3)	20 (5.0)	104 (26.1)	125 (31)	125 (31.3)
Availability of adequate rooms for service	12 (3.0)	18 (4.5)	26 (6.5)	129 (32)	214 (53.6)
Coffee ceremony after delivery in the health facility	80 (20.1)	15 (3.8)	40 (10)	48 (12)	216 (54.1)
Health workers Communication					
Information about plan of delivery, upcoming procedures and interventions and asked for consent	13 (3.3)	17 (4.3)	24(6.0)	107 (27)	238 (59.6)
Sufficient time devoted & information provided on obstetric related danger signs by health staffs	2 (.5)	18 (4.5)	19(4.8)	107 (27)	253 (63.4)
Health advices on new born care and breastfeeding	14 (3.5)	9 (2.3)	33 (8.3)	87 (22)	256 (64.2)
Health Care					
Capability of health staffs in identifying the patients’ problems and providing early response	0	14 (3.5)	18 (4.5)	145 (36)	222 (55.6)
Simplicity of obtaining drugs prescribed in the facility	4 (1.0)	8 (2.0)	26 (6.5)	131 (33)	230 (57.6)
Obtaining price free drugs for maternity service	10 (2.5)	17 (4.3)	15 (3.8)	75 (19)	282 (70.7)
Attitude of Health Workers					
Privacy during examination and delivery	3 (.8)	8 (2.0)	23 (5.8)	121 (30)	244 (61.2)
Respect of health staffs towards client	3 (.8)	5 (1.3)	13 (3.3)	101 (25)	277 (69.4)
Immediately knowing the condition of baby, maternal condition and seeing baby after delivery	0	0	14 (3.5)	85 (21)	300 (75.2)
Outcome of the service got during the stay	4 (1.0)	14 (3.5)	28 (7.0)	143 (36)	210 (52.6)
Complete services provided	1 (.3)	5 (1.3)	10 (2.5)	169 (42)	214 (53.6)

### Factors associated with clients’ satisfaction

Using Bartlett's Test of Sphericity and Kaiser-Meyer-Olkin (KMO) measure of sampling adequacy, factor analysis was performed on 31 variables that measure client satisfaction (Table 4). The KMO test shows that there are significant relationships among the perceived determinants of clients’ satisfaction as the KMO measure of sampling adequacy is 0.63 > 0.5, and Bartlett's Test of Sphericity with *p*-value <0.001 which is statistically significant showing that the variables are correlated highly enough to provide a reasonable basis for factor analysis. For factor analysis in varimax rotation convergence established after eighteen iterations and 66.7 % of the total variance was explained by the first twelve factors with eigenvalues greater than one (Table 5).

**Table 4:** Rotated Components and their Loadings Factor

Total variance explained				
Total	Factor label	Rotation Sums of Squared Loadings		
		Total	% of Variance	Cumulative %
1	Obtaining price free drugs for maternity service	2.473	7.495	7.495
2	Sufficient time devoted & information provided	2.387	7.233	14.728
3	Educational	2.177	6.598	21.326
4	Availability of transportation & communication	2.120	6.425	27.751
5	complete services provided	1.924	5.829	33.580
6	The respect of health staffs	1.830	5.545	39.125
7	Adequate number of health staffs	1.794	5.437	44.562
8	Easily obtaining the drugs	1.604	4.862	49.424
9	Health advices	1.552	4.703	54.127
10	Drugs and supplies	1.528	4.630	58.757
11	Adequate rooms for service	1.393	4.222	62.979
12	Age of clients	1.221	3.699	66.677

**Table 5:** Kaiser-Meyer-Olkin and Bartlett’s Test

KMO and Bartlett's test		
Kaiser-Meyer-Olkin measure of sampling adequacy.		.631
Bartlett's test of sphericity	Approx. Chi-Square	3645.481
	df	528
	Sig.	.000

Checking for the overall significance of regression model, the model chi-square has a value of 114.78 and a probability of *p*-value (<0.001) (Table 6). This shows that the final model has a good-fit indicating that the predictor variables do have a significant effect on the dependent variable i.e., overall client’s satisfaction.

**Table 6:** Summary statistics of the likelihood ratio test

Model	Model fitting criteria	Likelihood ratio test		
	−2 Log likelihood	Chi-Square	Df	Sig.
Null model (intercept only)	290.108	114.777	29	.000
Final model	175.331			

Thus, the results of linear regression analysis depicted that availability of drugs and supplies, health workers’ communication, type of health care provided, and attitude of health workers were significantly associated with overall client’s satisfaction with EmONC services (Table 7).

**Table 7:** Factors associated with client satisfaction with EmONC services in selected health facilities in Jimma zone, April 2014 (*n* = 399)

Coefficients^a^					
Explanatory variable	Un standardized coefficients		Standardized coefficients	*P*-value	95.0 % CI for B
	B	Std. Error	Beta		
Satisfaction to availability of health staffs*	0.96	0.16	0.000	0.63	1.28
Satisfaction to availability of drugs and supplies	0.230	0.040	0.23	<0.001	(0.15, 0.34)
Satisfaction to health workers communication	0.213	0.039	0.21	<0.001	(0.14, 0.29)
Satisfaction to health care provided	0.338	0.059	0.34	<0.001	(0.22, 0.45)
Satisfaction to attitude of health workers	0.248	0.050	0.25	<0.001	(0.15, 0.35)

^a^Dependent Variable: Overall satisfaction with EmONC services

*constant

## Discussion

In this study, the overall clients’ satisfaction with EmONC was 79.4 %. This finding is comparable with the results of the studies conducted in selected public health facilities of Wolaita Zone (82.9 %), Jimma (77 %) and Assela Hospital (80.7 %). But, it is slightly higher than the finding of a study conducted in referral hospital of Amhara Region, Ethiopia (61.9%) [7–10]. This difference may be due to some improvements in health care systems from time to time, a difference in quality of services provided, expectation of mothers or the type of health facilities.

Clients’ satisfaction to obtaining price-free drugs for maternity service, clients’ respect by health workers, health advices on new born care and breast feeding, necessary information about obstetric related danger signs, and provision of privacy during vaginal examination were 70.7 %, 69.4 %, 64.2 %, 63.4 %, and 61.2 %, respectively. These results are greater than the findings of other similar studies such as the study conducted in Amhara Region, in which  clients’ privacy related satisfaction was 46.7 % and their cost related satisfaction was 52.7 %; and in a study in Pakistan, clients’ satisfaction to provision of knowledge and advice on maternity service was 46 % [7, 11]. The improvements in results of our study may be due to the currently established health care financing policy, which enabled clients to avail free delivery service at health institutions, especially at public health centers, and due to the type of health facilities as most of the hospital based services include some additional expensive drugs and services.

In Ethiopia, conducting ‘coffee ceremony’ during labour and delivery is the most common and community-based cultural practice, and the absence of such ceremony makes women dissatisfied. This was reflected in our study as the highest score for recorded complete dissatisfaction with non-existence of coffee ceremony after delivery in the health facility (20.1 %). The next recorded clients’ complete dissatisfaction was with lack of obstetric emergency transportation and communication (6.3 %). A study in selected health facilities of Wolaita zone, Southern Ethiopia showed maternal dissatisfaction on distance to health facility was (36.9 %) [8] which is in line with our present study finding.

The result of linear regression analysis revealed that four variables explained clients’ overall satisfaction with EmONC services. The first factor that affected the overall satisfaction is availability of drugs and supplies. Thus, a unit decrease in satisfaction to availability of drugs and equipment decreases overall client satisfaction by 0.23 unit (95 % CI:0.15, 0.31). This finding is similar with that of Wolaita Zone study, where availability of drugs and supplies influenced positively clients’ satisfaction [8].

Evidences show that communication between the client and health care provider has a significant impact on client’s satisfaction [12, 13]. The finding of our study shows that a unit decrease in satisfaction to communication with health workers decreases client’s overall satisfaction by 0.21 unit (95 % CI: 0.14, 0.29). This result is consistent with the findings of  the studies conducted at referral hospital in Amhara region and public health facilities of Wolaita zone, which showed higher satisfaction of clients with health care provider’s communication [7, 14].

Satisfaction with the health care provided and satisfaction with the attitude of health workers affected clients’ overall satisfaction positively. A unit decrease in satisfaction with the health care provided and a unit decrease in satisfaction with the attitude of health care workers decreases clients’ overall satisfaction by 0.34 unit (95 % CI: 0.22, 0.45) and 0.25 unit (95 % CIP: 0.15,  0.35), respectively. This result is also in line with that of the Amhara region referral hospital study, in which attitude of the health workers had a relatively higher satisfaction score [7]. Hence the current result could be an updated evidence from client’s perspective towards an efforts being made to make health institutions client oriented in Ethiopia.

### Limitation

Potential recall bias due to long time lapse after childbirth couldn’t be ruled out.

## Conclusions

The findings of this study depict that the availability of minimum standards of health facility infrastructure, human resources, supplies, drugs and equipment are below the clients’ expectations. This gap could result in deceasing maternal service utilization for key life-saving actions, which in turn impede reducing maternal and new born mortalities. Measuring and monitoring clients’ satisfaction with EmONC services is not an end in itself. It is a means to improve service to the public and program performance in general. Clients’ satisfaction measurement provides invaluable information for responsive and effective client consultation. Therefore, improving the quality of service as per clients’ need requires the contribution of every stakeholder, including the government sectors, Non-Governmental Organizations (NGOs), and the communities themselves.

## Abbreviations

CI: Confidence Interval
EDHS: Ethiopian Demographic and Health Survey
EmONC: Emergency Obstetric and Newborn Care
ETB: Ethiopian Birr
FMOH: Federal Ministry of Health
ERC: Ethical Review Committee
JSI/L10K: John Snow Research & Training Institute Inc./the Last Ten Kilometers Project
NGOs: Non-Governmental Organizations
